# How and Why the Choice of Success Criteria Can Impact Therapy Service Delivery: A Worked Example From a Psychological Therapy Service for Anxiety and Depression

**DOI:** 10.32872/cpe.10237

**Published:** 2023-12-22

**Authors:** Mark H. Wheeler, Sheina Orbell, Tim Rakow

**Affiliations:** 1 Department of Psychology, University of Essex, Colchester, United Kingdom; 2 Department of Psychology, Institute of Psychiatry, Psychology and Neuroscience, King’s College London, London, United Kingdom; Philipps-University of Marburg, Marburg, Germany

**Keywords:** Increasing Access to Psychological Therapy (IAPT), therapy outcomes, clinically significant change, reliable change, payment by results, anxiety, depression

## Abstract

**Background:**

Well-defined measures of therapeutic benefit are essential for evaluating therapies and services. However, there is no single gold standard for defining ‘successful’ outcomes. We therefore examined the potential impact of adopting different success criteria.

**Method:**

We analysed data for 7,064 patients undergoing psychological therapy in a single UK IAPT (Increasing Access to Psychological Therapy) Service, each patient being assessed for depression (PHQ-9) and anxiety (GAD-7) both at the start and end of treatment. Predictors of successful outcomes based on these measures were analysed separately for three different success criteria: based either on assessing clinically significant change, or reliable change, in depression and anxiety.

**Results:**

The choice of criteria had little bearing on which variables predicted successful outcomes. However, the direction of the relationship between initial PHQ-9 or GAD-7 score and outcome success reverses when the criteria used to judge success are changed: successful outcomes are less probable under clinically significant change criteria for patients entering the service with more severe depression and/or anxiety but are more probable for such patients under reliable change criteria.

**Conclusion:**

Relevant for clinicians, researchers, and policymakers, the choice of success criteria adopted can substantially change the incentives for patient selection into a therapy service. Our analysis highlights how the methods used to evaluate treatment outcomes could impact the priorities and organisation of therapeutic services, which could then impact on who is offered treatment. We recommend further investigations of success criteria in other conditions or treatments to determine the reproducibility of the effects we found.

## Highlights

- Changing the criteria for judging therapy success alters treatment incentives.
- The choice of success criteria changes which cases are likely to have successful outcomes.
- Incentives to treat a patient group are substantially affected by the success criteria chosen.

## Incentives in Healthcare Systems

Incentives abound in healthcare systems. Of course, the primary incentive is shaped by the goal of achieving good outcomes for patients. However, incentives can be created in numerous ways, and their (sometimes unintended) consequences are diverse. For example, one might expect that insurance-based systems and/or a culture of malpractice litigation encourage excessive use of diagnostic tests (e.g., additional testing with limited incremental predictive value) because the costs of testing are easily covered (by insurance companies) and extensive testing provides concrete evidence of due diligence in diagnosis (a defence against litigation). In the – mainly publicly funded – UK health system, ‘*payment by results*’ has become increasingly common (e.g., NHS England and NHS Improvement, 2017a) with the laudable goal of incentivising best practice to improve services and clinical outcomes, while also increasing efficiency (Horton, 2007; Taunt et al., 2015). However, anecdotes of weaknesses in such target-driven approaches are commonplace. These include removing wheels from trolleys to create ‘beds’ that meet targets designed to reduce patients’ waiting-times on trolleys (Bevan & Hood, 2006) and having patients wait *outside* a hospital in ambulances to meet a maximum 4-hour waiting target *in* Accident and Emergency departments (Watts & Donnelly, 2012). Nonetheless, there is no *a priori* reason why well-designed financial incentives should not be used to improve the treatment that patients receive.

In 2017, NHS England and NHS Improvement issued detailed guidance to support service commissioners and providers to implement an outcomes-based payment approach for the UK’s flagship (publicly funded) IAPT Service (*Increasing Access to Psychological Therapy*; NHS England and NHS Improvement, 2017b). This mandated the use of an outcomes-based payment model for IAPT services from 1 April 2018 onwards, consisting of both a basic service price component reflecting activity *and* an outcomes payment component based on quality indicators and patient outcomes. The analysis presented in this paper primarily relates to the clinical outcomes element that comprises 50% of the outcomes payment component (with the other half of this component being based on performance against nine other quality and outcome measures). Note, however, that the application our analysis is not restricted to situations where payment by results is applied; but rather, to any situation where one clinical outcome measure is chosen in place of another or is given priority over another measure when outcomes are evaluated.

To illuminate the potential impact of the incentive structure created by the choice of clinical outcome measures, we analyse the clinical outcomes for both Depression and Generalised Anxiety Disorder from an IAPT service *prior to* the introduction of payment by results (PBR). To assess depression, IAPT services routinely use the Patient Health Questionnaire PHQ-9 (Kroenke et al., 2001) and for Generalised Anxiety Disorder the seven question GAD-7 measure (Spitzer et al., 2006). A quantitative assessment of the outcome of treatment is based on comparing pre- and post-therapy scores on the relevant clinical scale. However, there are different ways that this can be done in order to define a ‘successful’ treatment outcome (e.g., see Richards & Borglin, 2011). By considering three possible success criteria, and examining what predicts successful treatment outcomes according to each criteria in several thousand patients, we illustrate how the choice of success criteria could affect the incentives for patient selection for treatment. This is important because when incentives change, behaviour often changes – though not necessarily as hoped for by those creating the incentive structure (Gneezy & Rustichini, 2000).

## Success Criteria in Psychological Therapy

Jacobsen and colleagues (Jacobson et al., 1984; Jacobson & Revenstorf, 1988; Jacobson & Truax, 1991) proposed two criteria to ascertain whether or not the change experienced by a patient/client is meaningful: *clinically significant change* (CSC) and *reliable change* (RC).

The notion of *clinical significance* (as distinct from statistical significance) in therapy has been conceptualised in various ways, including: the practical value of the effect of an intervention (Risley, 1970); an improvement in the client’s everyday functioning (Kazdin & Wilson, 1978); a return to normal levels of functioning (Kendall et al., 1999; Nietzel & Trull, 1988) which is indistinguishable from that of their peer group (Kazdin, 1977). Operationalizing such considerations via standardised clinical assessments, Jacobson and colleagues proposed that *clinical significance* can be determined by the client’s score at post-treatment falling within the range for the functional population as opposed to the dysfunctional one (at pre-treatment). However, this criterion does not take account of measurement error, which may therefore give rise to misinterpretation due to regression to the mean; and there can also be difficulties determining what cut-off score(s) should divide the functional and dysfunctional populations (Tingey et al., 1996a, 1996b; Wampold & Jenson, 1986)

Measurement error is better dealt with in measures of (*statistically*) *reliable change*, which seek to determine whether a change is large enough to be considered meaningful. Such measures assess pre-post changes in scores on a clinical assessment relative to the standard error of that assessment tool (reflecting its reliability and the variability of scores in the normal/functional population). A reliable change can be said to have occurred if the pre-post change represents a statistically reliable improvement (or deterioration). Thus, the *size of change*, rather than whether change takes the patient across a threshold (as with CSC) is what determines success. This has the advantage of recognising improvements in symptoms even if the patient’s scores remain within the dysfunctional range (Lunnen & Ogles, 1998).

In our analysis of treatment outcomes, we follow the implementations of CSC and RC used by Richards and Borglin (2011) for the PHQ-9 and GAD-7 measures (the tools for assessing depression and anxiety used by IAPT, and reflected in the clinical outcomes element of the IAPT PBR system). Additionally, we examine outcomes according to an IAPT recovery criteria. This is a variant of the CSC approach but specifies different threshold (cut-off) scores to those for Richards and Borglin’s CSC implementation. The cut-off scores for this IAPT recovery criteria match the guidance given to general practitioners (GPs) regarding who to refer to an IAPT service (Clark et al., 2009) and are therefore important for determining which patients enter the IAPT service, how long they remain in it, and when they leave. This guidance dated from the set-up of the first IAPT services, and therefore precedes the introduction of PBR to IAPT by several years.

## Method

### Data

The anonymous dataset analysed (*N* = 7,064) comprised all patient cases undergoing therapy in a single IAPT service between 01 January 2009 and 14 February 2012 for whom both initial (start-of-treatment) and final (end-of-treatment) scores were available for both the PHQ-9 and GAD-7 measures. The data were provided by the IAPT Service in question. Scores for PHQ-9 and GAD-7 were used to categorise each case according to the three success criteria under consideration: IAPT recovery, clinically significant change and reliable change criteria.

### Application of Success Criteria to the Data

It made little sense to analyse successful outcomes for patients who, because of their pre-treatment scores, could not achieve a criterion for ‘success’. Therefore, for each of the three success criteria that we considered (Table 1) a subset of the data was created containing only those patients that could (in principle) have a successful outcome to their treatment. The process of creating these three subsets is described below.

#### IAPT Recovery Criteria

IAPT services were set up to provide psychological therapy primarily for patients with anxiety disorders and/or depression that is *at least* moderate (Clark et al., 2009). Therefore, ‘recovery’ is classified as moving a service user from a score (at first appointment) that would identify them as suitable for GP-referral (PHQ-9 > 9 or GAD-7 > 7) to a score (at last appointment) that is too low to trigger GP-referral to the service (PHQ-9 < 10 and GAD-7 < 8). Thus, when a patient’s initial score is close to the threshold specified by the IAPT criteria, a small reduction in their score is sufficient for a ‘recovery’ classification, e.g., from 10 to 9 for PHQ-9, and from 8 to 7 for GAD-7. However, much larger changes are required for a recovery classification when a patient’s initial score is high (e.g., severe depression with PHQ-9 of 21) because for this classification the final score must fall below the specified threshold. Consequently, for the IAPT recovery criteria, we analysed cases with initial PHQ-9 scores above 9, or initial GAD-7 scores above 7 because these were the patients (*N* = 6,338) who *could* ‘recover’ on these criteria.

#### Clinically Significant Change (CSC) Criteria

Following the definition from Richards and Borglin (2011), a success under the CSC criteria for depression is when PHQ-9 is above 8 pre-treatment and then is below 9 post-treatment. GAD-7 scores were required to be above 9 at pre-treatment and below 10 post-treatment. Thus, the minimum changes for a ‘success’ classification on the CSC criteria are from 9 to 8 for PHQ-9, and from 10 to 9 for GAD-7. This means that ‘success’ cannot be defined by the CSC criteria when a patient’s initial score is already below the specified threshold (i.e., PHQ-9 below 9, GAD-7 below 10). Therefore, for CSC, we analysed cases with initial PHQ-9 scores above 8, or initial GAD-7 scores above 9 (*N* = 6,127).

#### Reliable Change (RC) Criteria

For an outcome to be defined as showing reliable improvement, Richards and Borglin (2011) calculated that the PHQ-9 had to improve by 6 points or more and the GAD-7 by 5 points or more. Because the PHQ-9 and GAD-7 scales start at zero, a reliable change cannot be observed when a patient’s initial score is smaller than the size of change specified by the RC criteria. Therefore, for the RC criteria, we analysed cases with an initial PHQ-9 score above 5 or an initial GAD-7 score above 4. For our joint analysis of success according to reliable change on both measures, reported below, only cases above *both* cut-offs (simultaneously) are included (*N* = 6,218).

**Table 1:** Criteria Applied for the Analyses of Outcomes Defined by PHQ-9 and GAD-7 Scores (Analyses Are Reported in Tables 2-4)

Success criteria	Starting criteria^a^	Criteria to achieve a successful intervention	Number of cases analysed [available]^b^
IAPT recovery	Case has either a PHQ-9 > 9 or a GAD-7 > 7	Must record final scores of PHQ-9 < 10 and GAD-7 < 8	6,293 [6,338]
Clinically significant change (CSC)	Case has either a PHQ-9 > 8 or a GAD-7 > 9	Must record final scores of PHQ-9 < 9 and GAD-7 < 10	6,184 [6,229]
Reliable change (RC)	Case has both a PHQ-9 > 5 and a GAD-7 > 4	Must improve PHQ-9 score by 6 points or more and improve the GAD-7 score by 5 points or more	6,170 [6,218]

^a^Starting criteria represent the minimum score(s) needed to allow for the possibility of success; if scores fall below the specified cut-offs it is impossible to achieve a successful outcome with these criteria. ^b^Some cases with complete data for PHQ-9 and GAD-7 scores could not be included in the regression analysis of predictors of success due to missing data for predictor variables. Missing data are for Deprivation, Age or Gender.

For all three of the assessment methods, we used the success on both affect scales considered in combination as criteria for being an overall success for the patient (Table 1).

### Data Analysis

Within each data subset, each patient’s outcome was coded for success (no vs. yes) according to the criteria for IAPT Recovery, CSC and RC. Next, using *SPSS* software, three analyses were conducted using binary logistic regression, one for each data subset. Each analysis used the same set of 10 predictor variables (see Table 2, 3, or 4) to determine the independent predictors of success (for each success criteria in turn). These variables are ones that had previously been found to predict engagement with treatment and/or final scores for PHQ-9 or GAD-7 within this patient cohort (Wheeler, 2018). For simplicity and transparency of reporting, PHQ-9 scores were re-coded into one of five categories: minimal (scores of 0-4), mild (5-9), moderate (10-14), moderately severe (15-19) and severe (20-27). Four categories were used for the GAD-7: minimal (0-4), mild (5-10), moderate (11-15) and severe (16-21). Non-dichotomous categorical variables were dummy coded. For these variables, the reference category (i.e., ‘baseline category’, coded ‘0’) is shown in Tables 2, 3, and 4, together with the other category (coded ‘1’) for each dummy variable.

**Table 2:** Successful Outcomes for IAPT Recovery Criteria by Patient Category, and Logistic Regression With Successful Outcome as the Dependent Variable

Predictor variable (Level)	Baseline characteristic	% patients in category	% success within category	*p* ^a^	Adjusted odds ratio (*OR*)	*df*	99% CI for adjusted *OR* (lower and upper limits)
Gender							
	Male	33.4	39.0	–	–	–	–
Female		66.6	38.9	.297	1.069	1	0.907 – 1.260
Age in bands	16-24	15.9	33.3	–	–	–	–
Age 25-34 years		21.9	38.2	.516	1.066	1	0.827 – 1.374
Age 35-44 years		23.8	37.9	.677	1.042	1	0.810 – 1.340
Age 45-59 years		26.9	39.1	.309	1.102	1	0.861 – 1.411
Age ≥ 60 years		11.6	49.8	.020	1.348	1	0.967 – 1.878
Deprivation decile^b^	Decile 1-2	9.9	33.0	–	–	–	–
Decile 3-4		11.0	38.4	.218	1.172	1	0.841 – 1.635
Decile 5-6		32.5	38.6	.535	1.069	1	0.810 – 1.413
Decile 7-8		27.3	41.7	.080	1.213	1	0.913 – 1.610
Decile 9-10		19.4	38.6	.326	0.891	1	0.660 – 1.205
Employment^c^	In work	33.1	46.2	–	–	–	–
Unemployed seeking		17.0	42.1	.014	0.812	1	0.652 – 1.011
Students		20.8	25.8	**< .001**	0.490	1	0.393 – 0.611
Long term sick		7.8	28.9	**< .001**	0.535	1	0.391 – 0.733
Not actively seeking		19.5	42.5	**.001**	0.726	1	0.566 – 0.930
Retired		1.0	43.1	.304	0.754	1	0.372 – 1.530
Not known/stated		0.8	18.4	**.005**	0.317	1	0.109 – 0.919
Referral source^c^	GP	53.2	38.9	–	–	–	–
Self (i.e., patient)		41.0	40.7	.728	1.021	1	0.873 – 1.196
Secondary care		2.3	28.1	.056	0.675	1	0.398 – 1.146
Other source		3.6	25.3	**< .001**	0.535	1	0.340 – 0.843
Referral history	New referral	79.8	39.9	–	–	–	–
Re-referral		20.2	34.9	.381	.936	1	0.771 – 1.136
Psychotropic Medication	Not Prescribed	5.9	39.0	–	–	–	–
Prescribed, not taking		50.8	36.0	.853	1.024	1	0.740 – 1.416
Prescribed, taking		40.1	42.3	.596	1.070	1	0.771 – 1.484
Unknown/declined to say		3.3	44.2	.150	1.327	1	0.800 – 2.202
Initial PHQ-9^c^							
Minimal		2.4	68.4	**< .001**	4.923	1	2.923 – 8.260
Mild		10.9	61.5	**< .001**	3.763	1	2.834 – 4.995
Moderate		26.7	48.6	**< .001**	2.322	1	1.859 – 2.901
Moderately severe		32.0	35.3	**< .001**	1.591	1	1.591 – 1.955
	Severe	29.0	22.9	–	–	–	–
Initial GAD-7^c^							
Minimal		1.6	58.0	**< .001**	3.075	1	1.710 – 5.528
Mild		20.4	55.7	**< .001**	2.229	1	1.796 – 2.766
Moderate		35.8	41.9	**< .001**	1.448	1	1.211 – 1.730
	Severe	42.3	27.6	–	–	–	–
Engagement^c^	Less than 25%	4.1	14.8	–	–	–	–
26 – 50%		31.2	22.9	**.007**	1.681	1	1.027 – 2.750
51 – 75%		39.2	42.0	**< .001**	4.462	1	2.749 – 7.244
76 – 100%		25.5	57.5	**< .001**	8.563	1	5.228 – 14.025

*Note. N* = 6,293 patients with initial PHQ-9 > 9 or initial GAD-7 > 7, as defined by the IAPT recovery starting criteria. CI = confidence interval. Model fit: -2LL = 7135.5, Nagelkerke *R*^2^ = .249, χ^2^(32, *N* = 6,293) = 1273.9, *p* < .001.

^a^*p*-values for significant differences (α = .01) from the baseline category are shown in bold face type. ^b^Predictor variable is significant, *p* < .01. ^c^Predictor variable is significant, *p* < .001.

Alpha was set to .01 to reduce the risk of capitalising on chance relationships (given the relatively large number of effects examined by each analysis), and as a conservative correction for the fact that there may be some dependence of observations that we could not remove from, or control for, in our anonymised dataset (e.g., two lines of data for a single individual representing two separate referral/treatment episodes; patients referred from the same GP surgery where we cannot rule out effects due to surgery-specific referral practices). Missing data were rare. If data were missing for variables included in an analysis, the case was excluded from that analysis. These exclusions never exceeded 0.8% of cases (see Table 1). To determine whether the conclusions are affected by our decision to analyse successful outcomes defined *jointly* by PHQ-9 and GAD-7 scores, we also conducted separate analyses for each affect scale using each of the three success criteria. For the sake of brevity, these six analyses are reported in Supplementary Materials (Tables S1-S6).

**Table 3:** Successful Outcomes for Clinically Significant Change (CSC) Criteria by Patient Category, and Logistic Regression With Successful Outcome as the Dependent Variable

Predictor variable (Level)	Baseline characteristic	% patients in category	% success within category	*p* ^a^	Adjusted odds ratio (*OR*)	*df*	99% CI for adjusted *OR* (lower and upper limits)
Gender	Male	33.5	40.2	–	–	–	–
Female		66.5	39.5	.621	1.032	1	0.875 – 1.217
Age in bands	16-24	16.0	35.3	–	–	–	–
Age 25-34 years		21.8	38.9	.990	0.999	1	0.776 – 1.286
Age 35-44 years		23.8	38.8	.809	0.977	1	0.760 – 1.256
Age 45-59 years		27.0	39.4	.978	1.003	1	0.784 – 1.283
Age ≥ 60 years		11.4	50.3	.057	1.279	1	0.916 – 1.784
Deprivation decile	Decile 1-2	10.0	34.8	–	–	–	–
Decile 3-4		10.9	39.1	.297	1.144	1	0.821 – 1.593
Decile 5-6		32.4	39.0	.804	1.027	1	0.779 – 1.354
Decile 7-8		27.3	42.3	.163	1.164	1	0.879 – 1.542
Decile 9-10		19.3	40.2	.464	0.918	1	0.681 – 1.239
Employment^b^	In work	33.1	47.8	–	–	–	–
Unemployed seeking		16.9	41.9	**.001**	0.745	1	0.597 – 0.929
Students		21.0	27.0	**< .001**	0.485	1	0.389 – 0.603
Long term sick		7.8	28.3	**< .001**	0.473	1	0.345 – 0.649
Not seeking		19.3	43.0	**< .001**	0.704	1	0.549 – 0.903
Retired		1.0	42.9	.170	0.684	1	0.335 – 1.396
Not known/Stated		0.8	22.0	.012	0.380	1	0.141 – 1.024
Referral source^b^	GP	53.2	39.9	–	–	–	–
Self (i.e., patient)		40.8	41.3	.949	1.004	1	0.857 – 1.176
Secondary care		2.4	26.5	**.007**	0.571	1	0.335 – 0.972
Other source		3.6	27.4	**.002**	0.591	1	0.380 – 0.919
Referral history	New referral	79.7	40.8	–	–	–	–
Re-referral		20.3	35.5	.415	0.940	1	0.775 – 1.142
Psychotropic Medication	Not Prescribed	5.9	38.4	–	–	–	–
Prescribed, not taking		51.1	36.4	.437	1.104	1	0.796 – 1.531
Prescribed, taking		39.7	43.7	.140	1.208	1	0.868 – 1.681
Unknown/declined to say		3.3	47.3	.012	1.638	1	0.985 – 2.723
Initial PHQ-9^b^							
Minimal		1.6	68.6	**< .001**	5.678	1	3.085 – 10.449
Mild		10.1	63.5	**< .001**	4.333	1	3.243 – 5.790
Moderate		27.1	49.8	**< .001**	2.552	1	2.044 – 3.185
Moderately severe		31.6	37.4	**< .001**	1.770	1	1.443 – 2.171
	Severe	29.5	23.2	–	–	–	–
Initial GAD-7^b^							
Minimal		1.9	60.2	**< .001**	2.510	1	1.447 – 4.354
Mild		18.7	53.0	**< .001**	1.806	1	1.451 – 2.248
Moderate		36.4	44.0	**< .001**	1.377	1	1.154 – 1.643
	Severe	43.0	29.5	–	–	–	–
Engagement^b^	Less than 25%	4.1	15.0	–	–	–	–
26 – 50%		31.2	23.6	**.003**	1.762	1	1.079 – 2.880
51 – 75%		39.4	43.0	**< .001**	4.657	1	2.872 – 7.552
76 – 100%		25.4	58.4	**< .001**	8.912	1	5.445 – 14.587

*Note. N* = 6,184 patients with initial PHQ-9 > 8 or initial GAD-7 > 9, as defined by the CSC starting criteria. CI = confidence interval. Model fit: -2LL = 7083.0, Nagelkerke *R*^2^ = .243, χ^2^(32, *N* = 6,184) = 1226.4, *p* < .001

^a^*p*-values for significant differences (α = .01) from the baseline category are shown in bold face type. ^b^Predictor variable is significant, *p* < .001.

**Table 4:** Successful Outcomes for Reliable Change (RC) Criteria by Patient Category, and Logistic Regression With Successful Outcome as the Dependent Variable

Predictor variable (Level)	Baseline characteristic	% patients in category	% success within category	*p* ^a^	Adjusted odds ratio (*OR*)	*df*	99% CI for adjusted *OR* (lower and upper limits)
Gender	Male	33.8	36.3	–	–	–	–
Female		66.2	36.6	.329	1.063	1	0.904 – 1.250
Age in bands	16-24	15.9	31.7	–	–	–	–
Age 25-34 years		22.1	36.1	.519	1.065	1	0.829 – 1.367
Age 35-44 years		23.6	37.3	.732	1.034	1	0.806 – 1.325
Age 45-59 years		27.1	37.2	.903	0.989	1	0.775 – 1.261
Age ≥ 60 years		11.2	41.1	.148	1.205	1	0.865 – 1.678
Deprivation decile	Decile 1-2	10.1	33.2	–	–	–	–
Decile 3-4		11.1	37.7	.187	1.178	1	0.855 – 1.623
Decile 5-6		32.6	36.6	.583	1.059	1	0.809 – 1.385
Decile 7-8		27.0	37.6	.358	1.103	1	0.838 – 1.452
Decile 9-10		19.2	35.7	.484	0.924	1	0.690 – 1.237
Employment^b^	In work	33.2	42.6	–	–	–	–
Unemployed seeking		17.2	36.0	**< .001**	0.737	1	0.591 – 0.918
Students		20.9	29.8	**< .001**	0.500	1	0.404 – 0.620
Long term sick		7.9	29.2	**< .001**	0.560	1	0.412 – 0.762
Not seeking		19.1	37.5	**< .001**	0.712	1	0.557 – 0.912
Retired		1.0	41.9	.594	0.864	1	0.426 – 1.751
Not known/Stated		0.8	16.3	**.004**	0.312	1	0.109 – 0.892
Referral source	GP	53.5	36.6	–	–	–	–
Self (i.e., patient)		40.6	37.6	.396	1.053	1	0.901 – 1.230
Secondary care		2.4	34.7	.558	0.894	1	0.547 – 1.461
Other source		3.5	25.5	.003	0.594	1	0.379 – 0.928
Referral history^b^	New referral	79.8	36.7	–	–	–	–
Re-referral		20.2	32.2	**.003**	0.802	1	0.662 – 0.972
Psychotropic Medication	Not Prescribed	5.8	40.2	–	–	–	–
Prescribed, not taking		51.1	36.2	.115	0.822	1	0.596 – 1.133
Prescribed, taking		39.8	35.9	.179	0.844	1	0.609 – 1.168
Unknown/declined to say		3.3	43.3	.381	1.185	1	0.720 – 1.950
Initial PHQ-9^b^							
Mild^c^		13.2	21.1	**< .001**	0.395	1	0.294 – 0.530
Moderate		26.3	34.4	.014	0.814	1	0.656 – 1.010
Moderately severe		31.2	41.1	.640	1.035	1	0.856 – 1.252
	Severe	29.3	40.6	–	–	–	–
Initial GAD-7^b^							
Mild^c^		22.5	23.2	**< .001**	0.456	1	0.364 – 0.572
Moderate		34.8	38.2	.038	0.870	1	0.733 – 1.034
	Severe	42.7	42.2	–	–	–	–
Engagement^b^	Less than 25%	4.1	13.4	–	–	–	–
26 – 50%		31.2	21.4	**.001**	1.896	1	1.146 – 3.136
51 – 75%		39.3	40.0	**< .001**	4.898	1	2.985 – 8.035
76 – 100%		25.4	53.5	**< .001**	8.740	1	5.284 – 14.454

*Note. N* = 6170 patients with both initial PHQ-9 > 5 and initial GAD-7 > 4, as constrained by the minimum size of RC. Model fit: -2LL = 7234.9, Nagelkerke *R*^2^ = .178, χ^2^(30, *N* = 6170) = 859.7, *p* < .001

^a^*p*-values for significant differences from the baseline category are shown in bold face type, though only when the overall effect for the variable is also significant (α = .01). ^b^Predictor variable is significant, *p* < .001. ^c^*Mild* was the lowest category analysed for PHQ-9 depression and GAD-7 anxiety because a successful outcome on the RC criteria cannot be achieved for patients with *minimal* depression or anxiety. This is because any initial score in the *minimal* category is already too low to allow for the size of reduction that the RC criteria require for a successful outcome.

## Results and Discussion

Tables 2, 3 and 4 summarise each analysis of the predictors of success for the IAPT, CSC and RC success criteria, respectively, together with descriptive statistics for the distribution of patient characteristics and rates of successful outcomes across the levels of each predictor. These analyses suggest that there is ‘nothing unusual’ about the patient population that we have analysed. Consistent with other analyses of IAPT service populations, women outnumber men by a ratio of 2-to-1, and uptake rates are not particularly high among older individuals (Clark, 2018). Also consistent with previous analyses (e.g., Gyani et al., 2013) those who attend a higher proportion of the treatment sessions that they are offered have substantially better outcomes (see the ‘engagement’ predictor in Tables 2, 3, and 4). Perhaps unsurprisingly, some groups *not* in employment (e.g., long-term sick) are less likely to have a successful outcome, as is also the case in some analyses for those whose referral to the service did not originate from primary or community healthcare services.

**Figure 1:**
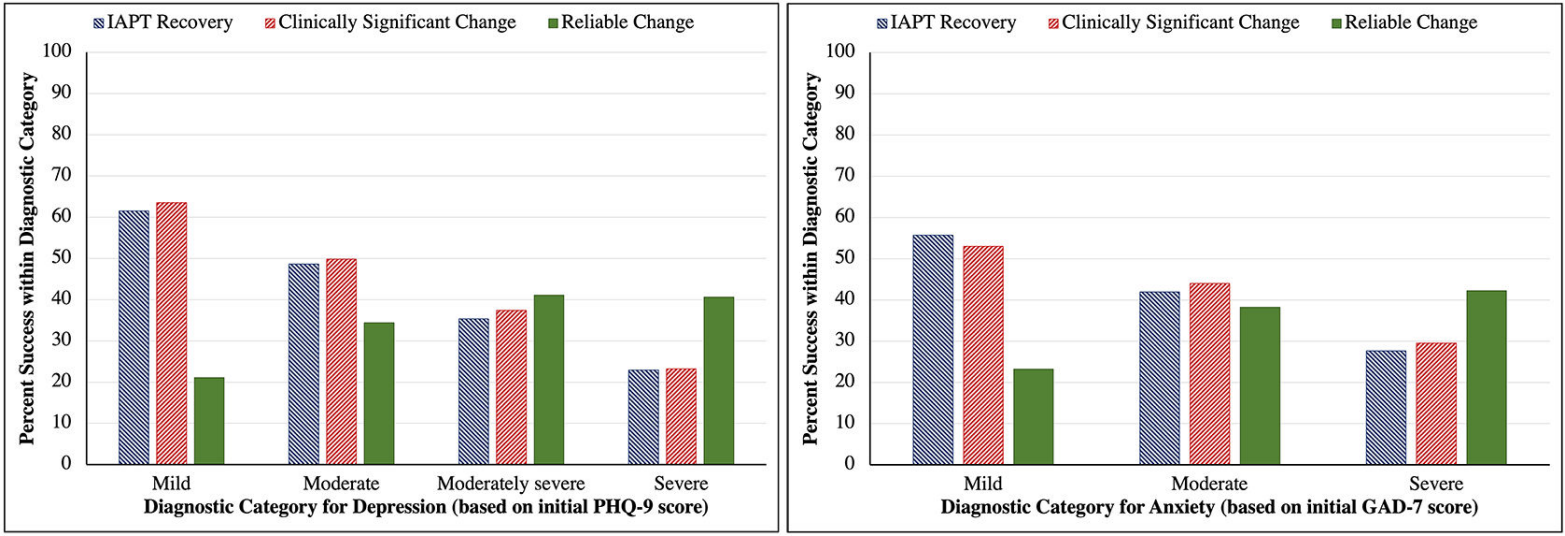
Success Rate by Diagnostic Category for Each of Three Success Criteria, for (a) Depression [left] and (b) Anxiety [right] *Note.* Minimal category not included because it is not examined in the analysis of the Reliable Change criteria.

Importantly, each of Tables 2, 3 and 4 show that both initial PHQ-9 scores (for depression) and initial GAD-7 scores (for anxiety) significantly predict successful outcomes. This is true for each of the three success criteria. However, as seen by comparing treatment success rates for each level of depression or anxiety across Tables 2, 3, and 4, the *direction* of effect is *not* the same for all three criteria. Figure 1 illustrates this pattern of effects. For the IAPT recovery criteria, success rates are progressively lower for more severe levels of depression or anxiety: effects that are large, statistically significant, and follow approximately linear progressions across different levels of PHQ-9 and GAD-7 (Table 2). An equivalent pattern and similar size of effect is seen for the CSC criteria: with the lowest rates of success found among those with severe depression and severe anxiety (Table 3). In contrast, this pattern is reversed when success is defined by RC: success rates are highest for those with severe depression or anxiety and lowest for those with mild depression or anxiety (Table 4). These effects are not so large as the equivalent ones for the IAPT recovery and CSC criteria. Nonetheless, the effects are statistically significant, both for depression and anxiety, and reveal that the recovery rate approximately doubles between the mild and severe categories on either the PHQ-9 or GAD-7.

The analyses reported in the Supplementary Materials confirm that initial scores for the affect measures also predict successful outcome when these outcomes are analysed separately for depression and anxiety. The direction of these effects reported in the Supplementary Materials (Tables S1-S6) match those described in the previous paragraph. Thus, consistent with the conclusions drawn from the analyses reported in Tables 2, 3, and 4 and illustrated in Figure 1, higher initial PHQ-9 scores are associated with a *lower* chance of successful outcome for depression when assessed on the IAPT recovery or CSC criteria, but a *higher* chance of successful outcomes for depression when assessed via RC criteria (Tables S1-S3). Likewise, the chances of a successful outcome for anxiety on the IAPT recovery or CSC criteria *reduce* as initial GAD-7 scores increase, but *increase* for the RC criteria as initial GAD-7 scores increase (Tables S4-S6). Moreover, the effects reported in the *Supplementary Materials* are always descriptively stronger than the corresponding effects reported in Tables 2, 3, and 4. That is, when predicting treatment success for depression (Tables S1-S3) the odds ratios (*OR*s) for each level of the PHQ-9 are further from 1 (i.e., ‘no effect’) than the corresponding *OR*s reported for the PHQ-9 in Tables 2, 3 and 4. And likewise, when predicting treatment success for anxiety (Tables S4-S6) the *OR*s for each level of the GAD-7 are further from 1 than the corresponding *OR*s reported for the GAD-7 in Tables 2, 3 and 4. From this we infer that the findings reported in Tables 2, 3, and 4 are *not* an artefact of analysing success criteria based jointly on outcomes for depression and anxiety. Indeed, reporting analyses based on such joint criteria may have resulted in a conservative illustration of the general patterns that we find.

We assume that the implications of these findings are clear with respect to the incentives for which patients are prioritised for treatment, irrespective of whether those incentives are created by the goals that the service sets for itself, or derive from another source such as via payment by results (PbR). The choice of success criteria could impact on which patients are most worthwhile treating. When success is defined by the *principles of clinically significant change* (IAPT recovery and CSC criteria) the chances of success are better for those whose depression and/or anxiety is not so severe. If these criteria are adopted, the service is incentivised to treat the less severe cases and to encourage those with more severe depression and/or anxiety to seek treatment outside the service. When success is defined according to the *principles of statistically reliable change*, the chances of success are better for those whose condition is severe. If such criteria are adopted, this incentivises treatment of more severe cases, and therefore dis-incentivises taking the relatively less severe cases into the service. It is *not* necessary for service providers to be consciously aware of this incentive structure for the incentives to have this effect: changes in patterns of referral, acceptance into the service, and extension of treatment provision for those most likely to achieve ‘success’ (however defined) can all happen gradually (perhaps imperceptibly so) following a simple ‘trial-and-improvement’ or stimulus-reward mechanism.

To illustrate how such a mechanism might play out in a specific context, we consider one of the changes to service funding that occurred subsequent to the period in which our data were recorded. The Guidance from NHS England and NHS Improvement (2017b) for outcomes-related payments to IAPT services (which came into effect on 01 April 2018) gave precedence to statistically reliable change in payments to IAPT services for the *clinical outcomes* component. Payment that rewards the clinical outcome for a patient was *only* made if there was statistically reliable improvement. There was, however, some regard for the principles of clinically significant change in these payments because the full payment was only made if the patient’s score drops below the cut-off for the IAPT recovery criteria. Failing that, payment was proportional to the degree of movement towards recovery. Given our analysis reported in this paper, such a PbR structure that emphasises reliable change seems to provide an incentive to prioritise treatment for those with more severe levels of depression and anxiety.^1^1Additionally, the third largest component of the payment model, *reducing disability and improved wellbeing* (10% weighting), also has payments linked to statistically reliable improvement.

Such incentives may be entirely reasonable: prioritising intervention for those whose conditions are most severe may bring the greatest reduction in the ‘global burden’ (for individuals, on their families, and to the economy) associated with mental ill health; and financial rewards to a service for treating these patients may offset the costs of treating these patients who are likely to have longer-than-average programmes of treatment. That said, we note that the IAPT Programme was set up to provide a readily accessible service to those with at least moderate depression and/or anxiety disorders – and not necessarily to treat the most severe cases of these conditions (Clark et al., 2009). What our analysis illustrates is that the choice of success criteria – for whatever reason they are adopted – can be important for which patients a service targets and therefore treats.

It is, of course, a limitation that our analyses use a single dataset and focussed on only two clinical measures for two mental health conditions. We conjecture that the patterns we find arise from the principles that differentiate CSC from RC criteria, and should be apparent in other contexts. Nonetheless, further investigations should examine the reproducibility of our findings in other mental health conditions and for implementations of CSC and RC in clinical measures other than the PHQ-9 and GAD-7. Another area for future research is to examine whether and how patients’ individual therapy goals map onto CSC or RC criteria. For example, can patients’ goals be expressed in terms of changes or thresholds on clinical measures, how do those goals vary with a patient’s starting point, and by what process do patients set their goals?

When considering how our findings relate to the academic literature on PbR in mental health services, it surprised us how small that body of literature seems to be. To illustrate, a search of the PUBMED database for “payment by results” [in article] AND “mental health” [in title/abstract] yielded only 13 articles^2^2We are grateful to a reviewer for pointing us towards this literature. Our search, conducted in July 2023, identified one further article. However, this article was on homelessness, not mental health services, and its single reference to payment by results did not refer to mental health.. As best we could determine, all 13 articles had PbR *in UK* mental health services as their main focus. However, Mason et al. (2011) also examined what the UK NHS could learn from the experience of the small number of countries in which PbR for mental health services had been explored (Australia, Canada, New Zealand) or implemented (The Netherlands, USA). A few other articles also reflected on PbR in mental health in some of those countries (e.g., Tulloch, 2012) usually by drawing on Mason et al. (2011).

However, important for the analyses that we report, the ‘results’ in these PbR schemes were *service activity* not *clinical outcomes*. These PbR schemes set price tariffs for mental health services contingent on the features of the clinical populations being treated. Higher prices are set for patients whose treatment is judged likely to be costly (e.g., because their diagnosis means treatment will probably be resource-intensive). Though rather different to the outcome-based PbR that we have focussed on in this article, our findings may point to a potential additional complexity associated with activity-based PbR. If treatment stops when a ‘successful’ outcome is achieved, but otherwise may continue, the choice of success criteria should impact what resources are allocated to a given patient. This is because – as our analyses show – the choice of success criteria impacts how condition severity relates to a ‘successful’ treatment outcome. Under CSC criteria, the chances of success are better for patients whose condition is less severe, and therefore these are the patients *least* likely to receive extended (costly) treatment. Conversely, a service that aims for ‘success’ under RC criteria will likely deploy *more* resources to treat these same patients because it will be more difficult (and therefore take longer) to achieve a successful outcome for their patients whose condition is less severe. Thus, when designing an activity-based PbR scheme, assuming one success criteria or another could (perhaps should) impact what price tariffs are set. And when operating under an established activity-based PbR scheme, the success criteria that a service adopts (explicitly or implicitly) in its clinical practice could affect whether or not service funding reflects service costs.

As a general point of application, our analyses illustrate that the question ‘Which type of patients respond best to this treatment?’ is *not* a context free question. Crucially, the answer to that question can depend on what criteria are used to measure a ‘successful’ response to treatment. Specifically, whether a successful outcome is determined according to a threshold for a clinical outcome measure (e.g., CSC) or according to the extent of improvement in such a clinical outcome (e.g., RC) can determine whether it appears that treatment is more successful for patients with less severe, or more severe, symptoms. Our goal is *not* to argue that one success criterion is best, or that another is inappropriate. Rather, we offer this analysis to emphasise that because incentives affect behaviour, success criteria must be chosen carefully if a therapy service is to operate according to its stated goals.

## Supplementary Materials

The Supplementary Materials (see Wheeler et al., 2023) report analysis of the predictors of a successful treatment outcome, separately for each affect scale (PHQ-9 and GAD-7), and separately for each of the three success criteria. These six analyses, using logistic regression, serve as a 'check' on the conclusions from the three analyses that are reported in the article.



WheelerM. H.
OrbellS.
RakowT.
 (2023). Supplementary materials to "How and why the choice of success criteria can impact therapy service delivery: A worked example from a psychological therapy service for anxiety and depression"
[Additional analyses]. PsychOpen. 10.23668/psycharchives.13964
PMC1086368038357428

## Data Availability

Data are not publicly available due to the privacy policy of the Service in relation to patient data. For questions about the data or its analysis, please contact the corresponding author.

## References

[sp1_r1] Wheeler M. H. Orbell S. Rakow T. (2023). Supplementary materials to "How and why the choice of success criteria can impact therapy service delivery: A worked example from a psychological therapy service for anxiety and depression" [Additional analyses]. PsychOpen. 10.23668/psycharchives.13964 PMC10863680 38357428

